# Supplementary material for: Quantitative Textural and Rheological Data on Different Levels of Texture-Modified Food and Thickened Liquids Classified Using the International Dysphagia Diet Standardisation Initiative (IDDSI) Guideline
Source: Foods. 2023 Oct 13;12(20):3765. doi: 10.3390/foods12203765 (PMC10606379; doi:10.3390/foods12203765)
Supplement: Supplementary file 1 [file foods-12-03765-s001.zip › foods-2640306-Supplementary/foods-2640306-Figure S1.pdf]

Supplementary Materials

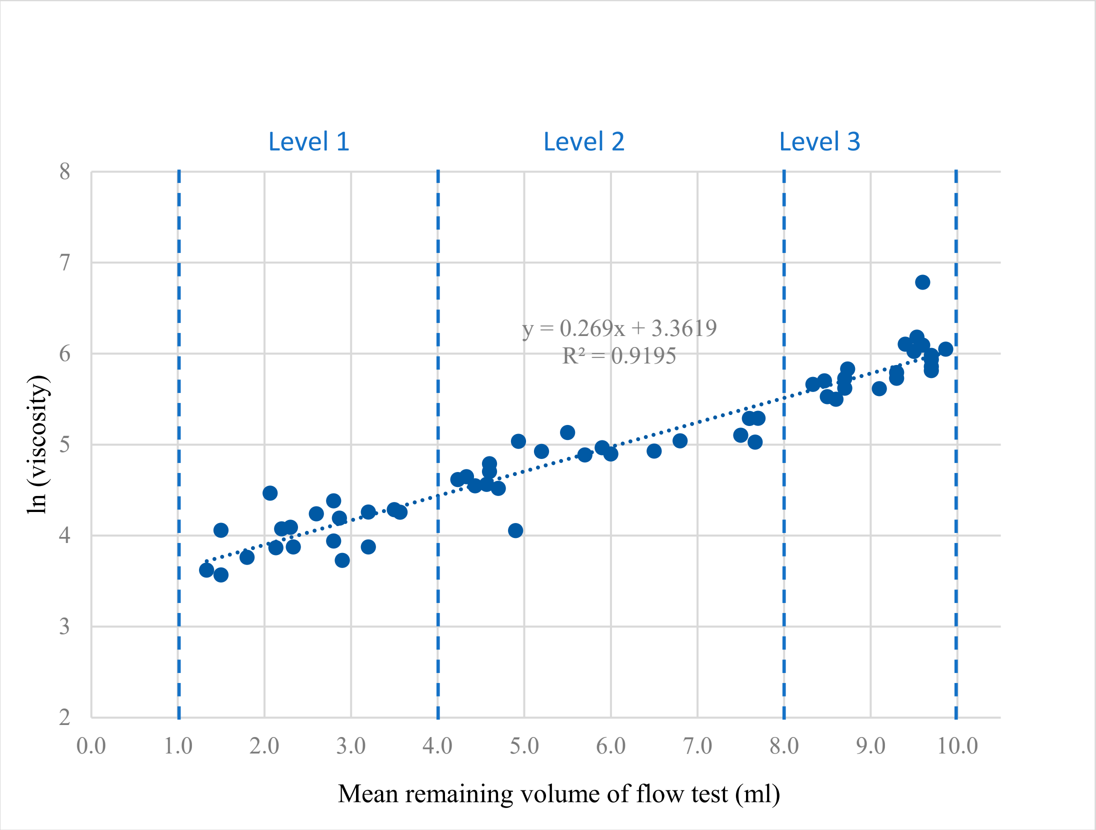

Figure S1. Correlation of remaining volume of flow test and natural logarithm of viscosity of different ingredients in different IDDSI levels. As level 0 and level 4 are beyond the test limit of syringe flow test, only samples of level 1 to 3 are included for the regression analysis.
