# Supplementary material for: Quantitative Textural and Rheological Data on Different Levels of Texture-Modified Food and Thickened Liquids Classified Using the International Dysphagia Diet Standardisation Initiative (IDDSI) Guideline
Source: Foods. 2023 Oct 13;12(20):3765. doi: 10.3390/foods12203765 (PMC10606379; doi:10.3390/foods12203765)
Supplement: Supplementary file 1 [file foods-12-03765-s001.zip › foods-2640306-Supplementary/foods-2640306-supplementary.pdf]

**Supplementary Materials**

**Table S1.** List of 41 food ingredients and 19 dishes for TPA in this study.

| Type | Fish and Meat | Vegetables | Fruits | High proteins food | High starch food |
|---|---|---|---|---|---|
| Ingredients | • Beef<br>• Chicken<br>• Dace paste<br>• Fish fillets<br>• Pork | • Black fungus<br>• Broccoli<br>• Carrot<br>• Chinese amaranth<br>• Chinese white cabbage<br>• Choy sum<br>• Eggplant<br>• Hairy gourd<br>• Iceberg lettuce<br>• Indian lettuce<br>• Mini Tientsin cabbage<br>• Shiitake mushroom<br>• Snow Fungus<br>• Tomato<br>• Wax gourd<br>• White radish | • Apple<br>• Kiwi<br>• Mango<br>• Orange<br>• Papaya<br>• Pineapple<br>• Watermelon | • Century egg<br>• Dried bean curd skin<br>• Dried octopus<br>• Dried shrimp<br>• Egg<br>• Tofu | • Chestnut<br>• Corn<br>• Green bean vermicelli<br>• Potato<br>• Pumpkin<br>• Rice<br>• Taro |
| Type | Fish | Meat | Condiments | Dessert | Others |
| Dishes or commercial products | • Fried dace with salted black beans<br>• Fried grouper<br>• Steamed snubnose pompano | • Barbecue pork<br>• Sweet and sour pork<br>• Trotter | • Garlic<br>• Peanut<br>• Salted black bean (Black bean sauce)<br>• Scallion oil | • Mooncake<br>• Sago<br>• Sesame dumpling | • Dried bonito<br>• Preserved Radish<br>• Steamed rice roll |

**Table S2.** List of 21 liquid food items for rheological test in this study.

| Type | Thickened by gum-based thickener | Thickened by corn starch | No thickener added |
|---|---|---|---|
| Food items | • Fish soup<br>• Pork soup<br>• Carrot juice<br>• Choy sum soup<br>• Corn soup<br>• Pumpkin soup<br>• Apple juice<br>• Mango juice<br>• Orange juice<br>• Papaya juice<br>• Pineapple juice<br>• Watermelon juice<br>• Soybean milk<br>• Coconut milk | • Black bean sauce<br>• Chu hou paste<br>• Coconut curry sauce<br>• Red fermented bean curd sauce | • Rice<br>• Peanut sauce<br>• Scallion oil |

**Table S3.** List of the methods to prepare food ingredients to different IDDSI levels.

| Type | Ingredients | IDDSI levels | | | |
|---|---|---|---|---|---|
| | | 7 | 6 | 5 | 4 (solid) |
| Fish and meat | Dace paste | • Cutting mud carp paste into 4cm diameter×1.5cm height<br>• Steaming with medium heat for 15 mins | • Steaming mud carp paste with medium heat for 15 mins<br>• Cutting into pieces smaller than 1.5cm×1.5cm×1.5cm | • Mixing mud carp paste with water in 4:1 ratio<br>• Steaming with medium heat for 15 mins<br>• Mincing into smaller than 0.4cm×0.4cm×0.4cm | • Blending level 6 samples with 3:1 water<br>• Filtering away excess water and lumps |
| | Fish fillets | • Pan-frying Pangasius fillets with medium heat for 5 mins<br>• Cutting into 4 cm diameter × 1.5 cm height | • Pan-frying Pangasius fillets with medium heat for 5 mins<br>• Cutting into pieces smaller than 1.5cm ×1.5cm×1.5cm | • Pan-frying Pangasius fillets with medium heat for 5 mins and adding some water to make it moist<br>• Mincing it into smaller than 0.4cm ×0.4cm×0.4cm | • Blending level 7 sample with water in 2:1 ratio<br>• Filtering away excess liquid and lumps |

| | | | | | |
|---|---|---|---|---|---|
| | Beef | • Cutting the lean beef into cubes<br>• Stir-frying for 2 mins with medium heat | • Cutting the beef flank steak into shred and shorter than 1.5cm<br>• Stir-frying for 2 mins with medium heat | • Stir-frying minced beef for 5 mins with medium heat and adding some water to make it moist | • Blending level 5 sample with water in 2:1 ratio<br>• Filtering excess liquid and lumps |
| | Chicken | • Cutting the chicken tenderloin into cube<br>• Boiling for 10 mins with medium heat | • Cutting the chicken tenderloin into shred and shorter than 1.5cm<br>• Boiling for 10 mins with medium heat | • Mincing the chicken tenderloin<br>• Boiling for 5 mins with medium heat | • Blending level 6 sample with water in 3:2 ratio<br>• Filtering excess liquid and lumps |
| | Pork | • Cutting pork rump meat into cube<br>• Boiling for 10 mins with medium heat | • Cutting pork rump meat into shred and shorter than 1.5cm<br>• Boiling for 10 mins with medium heat | • Steaming minced pork with water in 2:1 ratio for 10 mins with medium heat | • Blending level 5 samples with water in 2:1 ratio<br>• Filtering away excess liquid and lumps |
| Vegetable | Black fungus<br>**Level 4 is too sticky, so it is not recommended to be eaten individually* | • Soaking the black fungus in water for an hour<br>• Cutting into smaller pieces<br>• Steaming with medium heat for 15 mins | • Soaking the black fungus in water for an hour<br>• Cutting into pieces smaller than 1.5cm × 1.5cm × 1.5cm<br>• Steaming with medium heat for 15 mins | • Soaking the black fungus in water for an hour<br>• Mincing into pieces smaller than 0.4cm × 0.4cm × 0.4cm<br>• Steaming with medium heat for 15 mins | • Blending level 7 samples with water in 1:1<br>• Filtering away excess water and lumps |
| | Broccoli | • Cutting into smaller pieces<br>• Boiling with medium heat for 10 mins | • Cutting into smaller pieces<br>• Boiling with medium heat for 10 mins<br>• Cutting into pieces smaller than 1.5cm × 1.5cm ×1.5cm | • Cutting into smaller pieces<br>• Boiling with medium heat for 10 mins<br>• Mincing into pieces smaller than 0.4cm ×0.4cm ×0.4cm | • Blending cooked level 7 samples with water in 4:1 ratio<br>• Filtering away excess liquid and lumps |
| | Carrot | • Peeling away the skin<br>• Cutting into 4 cm diameter × 1.5 cm height<br>• Boiling for 10 mins with medium heat | • Peeling away the skin<br>• Cutting into pieces smaller than 1.5cm ×1.5cm×1.5cm<br>• Boiling for 25 mins with medium heat | • Peeling away the skin<br>• Mincing into smaller than 0.4cm×0.4cm×0.4cm<br>• Boiling for 20 mins with medium heat | • Blending level 6 samples with water in 2:1 ratio<br>• Filtering away excess liquid and lumps |

| | | | | | |
|---|---|---|---|---|---|
| | Chinese amaranth | • Cutting into 4cm diameter×1.5cm height<br>• Steaming with medium heat for 10 mins | • Cutting into 4cm diameter×1.5cm height<br>• Steaming with medium heat for 10 mins | • Cutting into 4cm diameter×1.5cm height<br>• Steaming with medium heat for 10 mins | • Blending level 7 samples |
| | Chinese white cabbage | • Cutting into two halves<br>• Boiling with medium heat for 10 mins | • Cutting into pieces smaller than 1.5cm × 1.5cm ×1.5cm<br>• Boiling with medium heat for 10 mins | • Mincing into pieces smaller than 0.4cm × 0.4cm × 0.4cm<br>• Boiling with medium heat for 5 mins | • Blending cooked level 7 samples with water in 10:1 ratio<br>• Filtering away excess liquid and lumps |
| | Choy sum | • Removing the lowest part of the stem<br>• Boiling with medium heat for 3 mins<br>• Cutting into a half | • Removing the lowest part of the stem<br>• Boiling with medium heat for 5 mins<br>• Cutting into pieces smaller than 0.75cm ×1.5cm ×1.5cm | • Removing the lowest part of the stem<br>• Mincing into pieces smaller than 0.4cm ×0.4cm ×0.4cm<br>• Boiling with medium heat for 3 mins | • Blending level 6 samples with water in 6:1<br>• Filtering away excess water and lumps |
| | Eggplant | • Cutting into 4cm diameter×1.5cm height<br>• Steaming with medium heat for 15 mins | • Removing the skin<br>• Cutting into pieces smaller than 1.5cm × 1.5cm ×1.5cm<br>• Steaming with medium heat for 10 mins | • Removing the skin<br>• Mincing into pieces smaller than 0.4cm×0.4cm×0.4cm<br>• Adding some water and steaming with medium heat for 15 mins | • Blending level 7 samples with water in 10:3 ratio<br>• Filtering away excess liquid and lumps |
| | Hairy gourd | • Removing the skin<br>• Cutting into 4cm diameter ×1.5cm height<br>• Steaming with medium heat for 15 mins | • Removing the skin<br>• Cutting into pieces smaller than 1.5cm × 1.5cm ×1.5cm<br>• Steaming with medium heat for 10 mins | • Removing the skin<br>• Mincing into pieces smaller than 0.4cm ×0.4cm ×0.4cm<br>• Adding some water and steaming with medium heat for 15 mins | • Blending level 7 samples with water in 10:3 ratio<br>• Filtering away excess liquid and lumps |
| | Iceberg lettuce | • Removing the core of the lettuce<br>• Boiling with medium heat for 5 mins<br>• Cutting into smaller pieces | • Removing the core of the lettuce<br>• Boiling with medium heat for 5 mins<br>• Cutting into pieces smaller than 1.5cm × 1.5cm ×1.5cm | • Removing the core of the lettuce<br>• Boiling with medium heat for 5 mins<br>• Mincing into pieces smaller than 0.4cm × 0.4cm × 0.4cm | • Blending cooked level 7 samples with water in 10:1 ratio<br>• Filtering away excess liquid and lumps |

| | | | | | |
|---|---|---|---|---|---|
| | Indian lettuce | • Cutting into pieces<br>• Boiling for 3 mins with medium heat | • Cutting into pieces smaller than 1.5cm ×1.5cm ×1.5cm<br>• Boiling for 5 mins with medium heat | • Mincing into pieces smaller than 0.4cm×0.4cm ×0.4cm<br>• Boiling for 5 mins with medium heat | • Blending level 5 samples with water in 2:1 ratio<br>• Filtering away excess liquid and lumps |
| | Mini Tientsin cabbage | • Cutting into smaller pieces<br>• Boiling with medium heat for 15 mins | • Cutting into pieces smaller than 1.5cm × 1.5cm ×1.5cm<br>• Boiling with medium heat for 15 mins | • Mincing into pieces smaller than 0.4cm ×0.4cm ×0.4cm<br>• Boiling with medium heat for 10 mins | • Blending cooked level 7 samples<br>• Filtering away excess liquid and lumps |
| | Shiitake mushroom<br>**Level 7 and 6 is too tough be broken apart by the side of fork* | • Soaking the mushrooms in warm water with a pinch of sugar for an hour<br>• Removing the stalk<br>• Steaming with medium heat for 15 mins | • Soaking the mushrooms in warm water with a pinch of sugar for an hour<br>• Removing the stalk<br>• Steaming with medium heat for 15 mins<br>• Cutting into pieces smaller than 1.5cm × 1.5cm × 1.5cm | • Soaking the mushrooms in warm water with a pinch of sugar for an hour<br>• Removing the stalk<br>• Steaming with medium heat for 15 mins<br>• Mincing into pieces smaller than 0.4cm × 0.4cm × 0.4cm | • Blending level 7 samples with water in 1:1<br>• Filtering away excess water and lumps |
| | Snow fungus<br>**Level 4 is too sticky, so it is not recommended to be eaten individually* | • Soaking for 2 hrs<br>• Cutting into smaller pieces<br>• Boiling with medium heat for 15 mins | • Soaking for 2 hrs<br>• Cutting into pieces smaller than 1.5cm × 1.5cm ×1.5cm<br>• Boiling with medium heat for 15 mins | • Soaking for 2 hrs<br>• Mincing into pieces smaller than 0.4cm ×0.4cm ×0.4cm<br>• Boiling with medium heat for 15 mins | • Blending cooked level 7 samples with water in 4:3 ratio<br>• Filtering away excess liquid and lumps |
| | Tomato | • Removing the skin and the seed<br>• Dividing 1 into 6 pieces<br>• Boiling for 2.5 mins with medium heat | • Removing the skin and the seed<br>• Cutting into pieces smaller than 1.5cm ×1.5cm×1.5cm<br>• Boiling for 2.5 mins with medium heat | • Removing the skin and the seed<br>• Mincing into smaller than 0.4cm×0.4cm×0.4cm<br>• Boiling for 2 mins with medium heat | • Blending level 6 samples<br>• Filtering away excess liquid and lumps |
| | Wax melon | • Removing the skin and the seed<br>• Cutting into 4cm diameter ×1.5cm height<br>• Steaming with medium heat for 15 mins | • Removing the skin and the seed<br>• Cutting into pieces smaller than 1.5cm × 1.5cm ×1.5cm<br>• Steaming with medium heat for 15 mins | • Removing the skin and the seed<br>• Mincing into pieces smaller than 0.4cm×0.4cm×0.4cm<br>• Adding some water and steaming with medium heat | • Blending cooked level 7 samples<br>• Filtering away excess liquid and lumps |

| | | | | | |
|---|---|---|---|---|---|
| | | | | for 10 mins | |
| | White radish | • Removing the skin of white radish<br>• Cutting into 4cm diameter×1.5cm height<br>• Boiling with medium heat for 10 mins | • Removing the skin of white radish<br>• Cutting into pieces smaller than 0.75cm × 0.75cm ×1.5cm<br>• Boiling with medium heat for 10 mins | • Removing the skin of white radish<br>• Mincing into pieces smaller than 0.4cm×0.4cm×0.4cm<br>• Boiling with medium heat for 15 mins | • Blending level 7 samples with water in 20:1 ratio<br>• Filtering away excess liquid and lumps |
| Fruit | Apple | • Removing the skin and the seeds<br>• Cutting into 4cm diameter×1.5cm height<br>• Boiling with medium heat for 5 mins<br>OR<br>• Cutting into pieces smaller than 0.75cm × 1.5cm ×1.5cm | • Removing the skin and the seeds<br>• Cutting into pieces smaller than 0.75cm × 1.5cm ×1.5cm<br>• Boiling with medium heat for 10 mins | • Removing the skin and the seeds<br>• Mincing into pieces smaller than 0.4cm×0.4cm×0.4cm<br>• Stir-frying with some water and medium heat for 5 mins | • Blending cooked level 7 samples<br>• Filtering away excess liquid and lumps |
| | Kiwi | • Cutting both ends off the kiwi and peel away the skin<br>• Cutting into 4cm diameter×1.5cm height | • Cutting both ends off the kiwi and peel away the skin<br>• Cutting into pieces smaller than 1.5cm × 1.5cm ×1.5cm | • Cutting both ends off the kiwi and peel away the skin<br>• Mincing into pieces smaller than 0.4cm×0.4cm×0.4cm | • Blending level 7 samples<br>• Filtering away excess liquid and lumps |
| | Mango | • Removing the skin<br>• Cutting into 4cm diameter ×1.5cm height | • Removing the skin<br>• Cutting into pieces smaller than 1.5cm × 1.5cm × 1.5cm | • Removing the skin<br>• Mincing into pieces smaller than 0.4cm × 0.4cm × 0.4cm | • Blending level 7 samples<br>• Filtering away excess liquid and lumps |
| | Orange<br>**Level 5 is too juicy, so it is not recommended to be eaten individually* | • Removing the fibrous part and seed<br>• Cutting into 4 cm diameter × 1.5 cm height | • Removing the fibrous part and seed<br>• Cutting into pieces smaller than 1.5cm ×1.5cm×1.5cm | • Removing the fibrous part and seed<br>• Mincing into pieces smaller than 0.4cm ×0.4cm ×0.4cm | • Blending level 6 samples and filtering away excess liquid and lumps |
| | Papaya | • Removing the fibrous part and seed<br>• Cutting into pieces and | • Removing the fibrous part and seed<br>• Cutting into pieces smaller | • Removing the fibrous part and seed<br>• Cutting into pieces smaller | • Blending level 6 samples and filtering away excess liquid and lumps |

| | | | | | |
|---|---|---|---|---|---|
| | | boiling for 15 mins with medium heat<br>• Cutting into 4 cm diameter × 1.5 cm height | pieces and boiling for 25 mins with medium heat<br>• Cutting into pieces smaller than 1.5cm ×1.5cm×1.5cm | pieces and boiling for 25 mins with medium heat<br>• Mincing into 0.4cm ×0.4cm×0.4cm | |
| | Pineapple | • Removing the skin of fresh pineapple<br>• Cutting into smaller pieces<br>OR<br>• Boiling with medium heat for 5 mins | • Removing the skin of fresh pineapple<br>• Cutting into pieces smaller than 1.5cm × 1.5cm ×1.5cm | • Removing the skin of fresh pineapple<br>• Mincing into pieces smaller than 0.4cm ×0.4cm ×0.4cm | • Blending cooked level 7 samples<br>• Filtering away excess liquid and lumps |
| | Watermelon | • Removing the skin and seed<br>• Cutting into 4cm diameter ×1.5cm height | • Removing the skin and seed<br>• Cutting into pieces smaller than 0.75cm × 1.5cm ×1.5cm | • Removing the skin and seed<br>• Mincing into pieces smaller than 0.4cm×0.4cm×0.4cm | • Blending cooked level 7 samples<br>• Filtering away excess liquid and lumps |
| High protein food | Century egg | • Boiling with medium heat for 5 mins<br>• Removing the shell and cut into two halves | • Boiling with medium heat for 5 mins<br>• Removing the shell and cut into pieces smaller than 1.5cm × 1.5cm ×1.5cm | • Boiling with medium heat for 5 mins<br>• Removing the shell and mince into pieces smaller than 0.4cm ×0.4cm ×0.4cm<br>• Adding some water to make it moist | • Blending cooked level 7 samples with water in 4:3 ratio<br>• Filtering away excess liquid and lumps |
| | Dried bean curd skin | • Soaking for 15 mins<br>• Cutting into smaller pieces<br>• Boiling with medium heat for 5 mins | • Soaking for 15 mins<br>• Cutting into pieces smaller than 1.5cm × 1.5cm ×1.5cm<br>• Boiling with medium heat for 5 mins | • Soaking for 15 mins<br>• Mincing into pieces smaller than 0.4cm ×0.4cm ×0.4cm<br>• Boiling with medium heat for 5 mins | • Blending cooked level 7 samples with water in 5:1 ratio<br>• Filtering away excess liquid and lumps |
| | Dried octopus<br>**Level 7 and 6 are too tough to be broken apart by the side of fork, nor pass the fork pressure test* | • Soaking dried octopus overnight<br>• Cutting into pieces smaller than 0.75cm × 0.75cm × 0.75cm<br>• Boil for 20 mins with medium heat<br>• | • Soaking dried octopus overnight<br>• Blending cooked level 7 sample | • Soaking dried octopus overnight<br>• Blending cooked level 7 samples with water in 4:5 ratio<br>• | |
| | Dried shrimp<br>**Level 4 is not smooth enough and lump* | • Soaking for 10 mins<br>• Stir-frying with medium heat<br>• Adding some water and boil | • Soaking for 10 mins<br>• Cutting into pieces smaller than 0.75cm × 1.5cm ×1.5cm<br>• Stir-frying with medium | • Soaking for 10 mins<br>• Blending into pieces smaller than 0.4cm×0.4cm×0.4cm<br>• Stir-frying with medium | • Blending level 7 samples with water in 4:3 ratio<br>• Filtering away excess liquid and lumps |

| | | | | | |
|---|---|---|---|---|---|
| | *together, so it is not recommended to be eaten individually* | for 10 mins<br>• | heat<br>• Adding some water and boil for 10 mins | heat<br>• Adding some water and boil for 10 mins<br>• | • |
| | Egg | • Boiling egg with cold water and medium heat for 12 mins<br>• Removing the shell and cut into half | • Beating one egg<br>• Frying the mixture with medium heat until it is cooked<br>• Cutting into pieces smaller than 1.5cm ×1.5cm×1.5cm | • Mixing one egg with one tablespoon of water or milk<br>• Frying the mixture with stirring on medium heat until it is cooked<br>• Mincing into pieces smaller than 0.4cm×0.4cm×0.4cm | • Mixing egg with water in 1:2 ratio<br>• Steaming for 15 mins with medium heat<br>• Filtering away excess liquid |
| | Tofu | • Cutting dried bean curd into 4 cm diameter × 1.5 cm height<br>• Boiling for 5 mins with medium heat | • Cutting hard bean curd into 1.5cm ×1.5cm×1.5cm<br>• Boiling for 5 mins with medium heat | • Mincing bean curd for steaming into 0.4cm ×0.4cm×0.4cm<br>• Boiling for 1.5 mins<br>• with medium heat | • Blending level 6 samples and filtering away the lumps<br>OR<br>• Using prepackaged tofu fa |
| High starch food | Chestnut<br>**Level 4 is too sticky, so it is not recommended to be eaten individually* | • Boiling for 20 mins with medium heat | • Boiling for 40 mins with medium heat<br>• Cutting into pieces smaller than 1.5cm × 1.5cm × 1.5cm<br>• | • Boiling for 20 mins with medium heat<br>• Mincing into pieces smaller than 0.4cm × 0.4cm × 0.4cm | • Blending level 7 samples with water in 3:2 water |
| | Corn | • Steaming ear of corn for 10 mins with medium heat<br>• Cutting off corn kernels but avoiding the tip cap | | • Mincing level 6 samples into pieces smaller than 0.4cm ×0.4cm ×0.4cm | • Blending level 6 samples with water in 2:1 ratio<br>• Filtering away excess liquid and lumps |
| | Green bean vermicelli | • Soaking for 10 mins and boil with medium heat for 3 mins<br>• Cutting into smaller pieces | • Soaking for 10 mins and boil with medium heat for 3 mins<br>• Cutting into pieces smaller than 1.5cm × 1.5cm ×1.5cm | • Soaking for 10 mins and boil with medium heat for 3 mins<br>• Mincing into pieces smaller than 0.4cm×0.4cm×0.4cm | • Blending level 7 samples with water in 1:1 ratio<br>• Filtering away excess liquid and lumps<br>• |
| | Potato<br>**Level 4 is too sticky, so it is not recommended to be eaten individually* | • Peeling away the skin<br>• Cutting into 4 cm diameter × 1.5 cm height<br>• Boiling for 10 mins with medium heat<br>• | • Peeling away the skin<br>• Cutting into pieces smaller than 1.5cm ×1.5cm×1.5cm<br>• Boiling for 25 mins with medium heat<br>• | • Peeling away the skin<br>• Mincing into smaller than 0.4cm×0.4cm×0.4cm<br>• Boiling for 15 mins with medium heat | • Blending level 6 samples with water in 2:1 ratio Filtering away excess liquid and lumps |

| | | | | | |
|---|---|---|---|---|---|
| | Pumpkin<br>**Level 4 is too sticky, so it is not recommended to be eaten individually* | • Removing the skin<br>• Mincing into pieces smaller than 0.4cm×0.4cm×0.4cm<br>• Adding some water and steaming with medium heat for 15 mins | • Cutting into pieces smaller than 0.75cm × 0.75cm ×1.5cm<br>• Steaming with medium heat for 10 mins<br>• | • Mincing into pieces smaller than 0.4cm×0.4cm×0.4cm<br>• Adding some water and steaming with medium heat for 5 mins<br>• | • Blending level 7 samples with water in 3.5:2 ratio<br>• Filtering away excess liquid and lumps |
| | Rice*<br>**Level 4 is too sticky so it is not recommended to be eaten individually* | • Cooking with water in 1:1.2 ratio | • Cooking with water in 1:1.5 ratio | • Cooking with water in 1:3 ratio | • Mincing into pieces smaller than 0.4cm ×0.4cm ×0.4cm<br>• Boiling with medium heat for 3 mins |
| | Taro<br>**Level 4 is too sticky, so it is not recommended to be eaten individually* | • Removing the skin<br>• Cutting into 4cm diameter×1.5cm height<br>• Steaming with medium heat for 10 mins | • Removing the skin<br>• Cutting into pieces smaller than 0.75cm × 0.75cm ×1.5cm<br>• Steaming with medium heat for 15 mins | • Removing the skin<br>• Mincing into pieces smaller than 0.4cm×0.4cm×0.4cm<br>• Steaming with medium heat for 10 mins | • Blending level 7 samples with water in 3:1 ratio<br>• Filtering away excess liquid and lumps |

**Table S4.** List of the methods to prepare dishes or commercial products to different IDDSI levels.

| Type | Dishes or commercial products | IDDSI levels | | | | |
|---|---|---|---|---|---|---|
| | | 7 | 6 | 5 | 4 | |
| | | | | | Puree | Shaped-soft meal |
| Fish | Fried dace with salted black beans | • Steaming the commercial fried dace with salted black bean for 10 mins with medium heat<br>• Removing the bone<br>• Cutting into smaller pieces | • Steaming the commercial fried dace with salted black bean for 10 mins with medium heat<br>• Removing the bone<br>• Cutting pieces smaller than 0.75cm ×0.75cm ×0.75cm | • Steaming the commercial fried dace with salted black bean for 10 mins with medium heat<br>• Removing the bone<br>• Mincing into pieces smaller than 0.4cm ×0.4ccm ×0.4cm | • Blending level 7 samples with water in 1:1 ratio and filter away excess water and lumps | • Blending level 7 samples with water in 1:2 ratio and 1% enzyme gellant<br>• Blending salted black bean with water in 1:2 ratio and 1% enzyme gellant<br>• Heating separately with stirring until the paste become liquid and pouring into shaped mold and set for 5 min |
| | Fried grouper | • Using the commercial fried grouper in sweet corn sauce | • Cutting the commercial fried grouper into smaller than 1.5cm ×1.5cm ×1.5cm | • Mince the commercial fried grouper and corn into pieces smaller than 0.4cm ×0.4cm ×0.4cm<br>• Serve in sweet corn sauce | | • Blending the fried grouper with water in 1:2 ratio and 1% enzyme gellant<br>• Blending the corn with water in 1:1 ratio and 1% enzyme gellant<br>• Heating separately with stirring until the paste become liquid<br>• Pouring into shaped mold and setting for 5 min<br>• Serving in sweet corn sauce |
| | Steamed snubnose pompano | • Steaming with 10ml oil and 10g salted black bean with medium heat for 15 mins and adding soy sauce<br>• Removing bones and cutting into smaller pieces | • Steaming with 10ml oil and 10g salted black bean with medium heat for 15 mins and adding soy sauce<br>• Removing bones and cutting into pieces smaller than 1.5cm × 1.5cm ×1.5cm | • Steaming with 10ml oil and 10g salted black bean with medium heat for 15 mins and adding soy sauce<br>• Removing bones and mincing into pieces smaller than 0.4cm×0.4cm×0.4cm | • Blending cooked level 7 samples with water in 7:1 ratio<br>• Filtering away excess liquid and lumps | • Blending cooked level 7 sample with water in 1:2 ratio and 0.8% enzyme gellant and<br>• Blending salted black bean with water in 1:2 ratio and 1% enzyme gellant<br>• Heating with stirring until the paste become liquid and pouring into shaped mold and set for 5 min<br>• Serving with level 3 black bean sauce |
| Meat | Barbecued pork | • Using the commercial | • Using the commercial barbecue | • Removing the outer skin of the | • Blending the commercial | • Blending the barbecue pork with water in 1:2 ratio, char siu sauce |

| | | | | | | |
|---|---|---|---|---|---|---|
| | | barbecue pork<br>• Cutting it into slide | pork<br>• Cutting it into pieces smaller than 0.75cm ×1.5cm ×1.5cm | commercial barbecue pork<br>• Steaming with medium heat for 15 mins<br>• Blending the commercial barbecue pork and add some water to make it moist | barbecue pork with water in 1:1 ratio and filtering away excess water and lumps | and 1% enzyme gellant<br>• Heating with stirring until the paste becoming liquid and pouring into shaped mold and set for 5 min to make the outer part of barbecue pork<br>• Blending the barbecue pork with water in 1:2 ratio and 0.8% enzyme gellant<br>• Heating with stirring until the paste becoming liquid and pouring on the moulded outer part of the barbecue pork and setting for 5 min |
| | Sweet and sour pork | • Using the commercial sweet and sour pork without bone | | • Mincing the commercial sweet and sour pork without bone into 0.4cm ×0.4cm×0.4cm | • Blending into puree with water in 4:3 ratio | • Blending the sweet and sour pork with water in 1:2 ratio and 0.8% enzyme gellant<br>• Blending the pineapple with water in 1:1 ratio and 0.8% enzyme gellant<br>• Blending the green pepper with water in 1:1 ratio and 0.8% enzyme gellant<br>• Heating separately with stirring until the paste becoming liquid and pouring into shaped mold and setting for 5 min<br>• Serving in sweet and sour sauce |
| | Trotter | • Removing the bone from commercial trotter<br>• Cutting the trotter into smaller pieces | • Removing the bone from commercial trotter<br>• Cutting the trotter into pieces smaller than 1cm ×1cm ×1cm | • Removing the bone from commercial trotter<br>• Mincing the trotter into pieces smaller than 0.4cm ×0.4cm ×0.4cm | • Blending the trotter with water in 2:1 ratio and filtering away excess water and lumps | • Blending the trotter with water in 1:2 ratio and 1% enzyme gellant<br>• Heating with stirring until the paste become liquid and pouring into shaped mold and set for 5 min<br>• Cutting it into slices and placing on bones (the bones are just for display) |

| | | | | | | |
|---|---|---|---|---|---|---|
| Condiments | Garlic | • Cutting into pieces smaller than 1.5cm × 1.5cm ×1.5cm<br>• Stir-frying with medium heat for 1.5 mins | | • Mincing into pieces smaller than 0.4cm×0.4cm×0.4cm<br>• Stir-frying with medium heat for 1.5 mins | • Blending garlic with water in 2:1 ratio<br>• Filtering away excess liquid and lumps<br>• Stir-frying with medium heat for 1.5 mins | |
| | Peanut | • Soaking with water for an hour<br>• Boiling with medium heat for an hour | | | • Blending level 7 with water in 8:5 ratio<br>• Filtering away excess water and lumps | |
| | Salted black bean | • Boiling for 5 mins with medium heat | | • Boiling for 5 mins with medium heat<br>• Cutting into pieces smaller than 0.4cm × 0.4cm × 0.4cm | • Blending cooked level 7 samples with water in 1:2 ratio | |
| | Scallion oil | • Cutting scallion into pieces smaller than 1.5cm × 1.5cm ×1.5cm<br>• Stir-frying with plenty of oil with medium heat for 1.5 mins | | • Mincing scallion into pieces smaller than 0.4cm×0.4cm×0.4cm<br>• Stir-frying with plenty of oil with medium heat for 1.5 mins | • Blending cooked level 7 samples with water in 10:7 ratio<br>• Filtering away excess liquid and lumps | |
| Dessert | Mooncake | • Cutting pre-packaged mooncake into pieces | | | | • Blending egg yolk with water in 1:2 ratio and 0.5% enzyme gellant<br>• Heating with stirring until the paste becoming liquid and pouring into shaped mold<br>• Blending lotus seed paste with water in 1:2 ratio and 0.8% enzyme gellant<br>• Heating with stirring until the paste becoming liquid<br>• Pouring into shaped mold, add shaped egg yolk and setting for 10 |

| | | | | | | |
|---|---|---|---|---|---|---|
| | | | | | | min |
| | Sago | • Bringing a pot of water to boil, add sago and boil for 10 mins<br>• Turning off heat and wait for 15 mins, drain water from the cooked sago | | | | |
| | Sesame dumplings | • Boiling pre-packaged black sesame dumpling with medium heat for 5 mins<br>• Serving in ginger sweet soup | | | | • Blending level 7 samples with water in 1:2 ratio and 1% enzyme gellant<br>• Heating with stirring until the paste becoming liquid<br>• Pouring into shaped mold and set for 5 min<br>• Serving in thickened ginger sweet soup |
| Others | Dried bonito<br>**Level 4 is not smooth enough and lump together, so it is not recommended to be eaten individually.* | • Soaking for 15 mins<br>• Removing bones<br>• Cutting into smaller pieces<br>• Boiling with medium heat for 10 mins | • Soaking for 15 mins<br>• Removing bones<br>• Cutting into pieces smaller than 1.5cm × 1.5cm ×1.5cm<br>• Boiling with medium heat for 10 mins | • Soaking for 15 mins<br>• Removing bones<br>• Mincing into pieces smaller than 0.4cm ×0.4cm ×0.4cm<br>• Boiling with medium heat for 10 mins | • Blending cooked level 7 samples with water in 1:1 ratio<br>• Filtering away excess liquid and lumps | |

| | | | | | | |
|---|---|---|---|---|---|---|
| | Preserved radish | • Soaking the preserved radish for 10 mins and steaming with medium heat for 10 mins<br>• Cutting the preserved radish into cubes | • Soaking the preserved radish for 10 mins and steaming with medium heat for 10 mins<br>• Cutting the preserved radish into pieces smaller than 0.5cm ×0.5cm ×0.5cm | • Soaking the preserved radish for 10 mins and steaming with medium heat for 10 mins<br>• Blending the preserved radish into pieces smaller than 0.4cm ×0.4cm ×0.4cm and adding some water to make it moist | • Blending the preserved radish with water in 1:2 ratio and filtering away excess water and lumps | • Soaking the preserved radish for 10 mins and steaming with medium heat for 10 mins<br>• Blending the preserved radish with water in 1:5 ratio and 1% enzyme gellant and heating with stirring until the paste become liquid and pour into shaped mold and setting for 5 min<br>• Cutting it into slices |
| | Steamed rice-roll | • Using the commercial steamed rice roll<br>• Cutting it into smaller pieces | • Using the commercial steamed rice roll<br>• Cutting it into pieces smaller than 1.5cm×1.5cm×1.5cm | | | • Blending the steamed rice roll with water in 1:2 ratio and 1.5% enzyme gellant<br>• Heating with stirring until the paste becoming liquid and pouring thin layer in a rectangular plate and setting for 5 min<br>• Rolling up the paste |

**Table S5.** List of the methods to prepare liquid food items to different IDDSI levels.

| Type | Ingredients | IDDSI levels | | | | |
|---|---|---|---|---|---|---|
| | | 4 (liquid) | 3 | 2 | 1 | 0 |
| Thickened by gum-based thickener | Fish soup | • Frying 650g grass carp fish with medium heat until the two sides become lightly golden yellow in colour<br>• Adding 2.5L boiling water and boiling for 30 mins<br>• Removing the bone of the fish and blending the fish<br>• Adding the fish back to the soup and boiling for 5 mins<br>• Filtering away the residue to get the fish soup | | | | |
| | | • Adding 4.2g thickener into 100ml fish soup | • Adding 1.4g thickener into 100ml fish soup | • Adding 0.7g thickener into 100ml fish soup | • Adding 0.4g thickener into 100ml fish soup | • Using 100ml fish soup |
| | Pork soup | • Cutting 400g pork lean meat into slices or cubes<br>• Boiling pork with 2.5L water and medium heat for 15 mins<br>• Blending boiled pork, adding back to the soup and boiling for 5 more mins<br>• Filtering away the residue to obtain pork soup | | | | |
| | | • Adding 4.2g thickener into 100ml pork soup | • Adding 2.1g thickener into 100ml pork soup | • Adding 1.4g thickener into 100ml pork soup | • Adding 0.7g thickener into 100ml pork soup | • Using 100ml pork soup |
| | Carrot juice | • Blending carrot with water in 1:1 ratio<br>• Filtering away the residue to obtain the 50% carrot juice | | | | |
| | | • Adding 2.8g thickener into 100ml 50% carrot juice | • Adding 1.4g thickener into 100ml 50% carrot juice | • Adding 1.0g thickener into 100ml 50% carrot juice | • Adding 0.7g thickener into 100ml 50% carrot juice | • Using 100ml 50% carrot juice |
| | Choy sum soup | • Boiling 750g choy sum with 2.5L water medium heat for 15 mins<br>• Blending boiled choy sum, adding back to the soup and boiling for 5 more mins<br>• Filtering away the residue to obtain choy sum soup | | | | |
| | | • Adding 4.2g thickener into 100ml choy sum soup | • Adding 1.4g thickener into 100ml choy sum soup | • Adding 1.0g thickener into 100ml choy sum soup | • Adding 0.7g thickener into 100ml choy sum soup | • Using 100ml choy sum soup |
| | Corn soup | • Boiling corn 600g with 2.5L water medium heat for 15 mins<br>• Blending boiled corn, adding back to the soup and boiling for 5 more mins<br>• Filtering away the residue to obtain corn soup | | | | |

| | | | | | | |
|---|---|---|---|---|---|---|
| | | • Adding 2.8g thickener into 100ml corn soup | • Adding 1.4g thickener into 100ml corn soup | • Adding 0.7g thickener into 100ml corn soup | • Adding 0.4g thickener into 100ml corn soup | • Using 100ml corn soup |
| | Pumpkin soup | • Cutting 1kg pumpkin into slices or cubes and adding into 2.5L water<br>• Boiling pumpkin with medium heat for 30mins<br>• Filtering away the residue to obtain pumpkin soup | | | | |
| | | • Adding 3.5g thickener into 100ml pumpkin soup | • Adding 1.4g thickener into 100ml pumpkin soup | • Adding 0.7g thickener into 100ml pumpkin soup | • Adding 0.4g thickener into 100ml pumpkin soup | • Using 100ml pumpkin soup |
| | Apple juice | • Adding 4.2g thickener into 100ml 100% apple juice without pulp | • Adding 2.1g thickener into 100ml 100% apple juice without pulp | • Adding 1.4g thickener into 120ml 100% apple juice without pulp | • Adding 0.7g thickener into 100ml 100% apple juice without pulp | • Using 100ml 100% apple juice without pulp |
| | Mango juice | • Blending 500g mango with 1L water<br>• Filtering the residue to obtain 33.3% mango juice | | | | |
| | | • Adding 4.2g thickener into 100ml 33.3% mango juice without pulp | • Adding 1.4g thickener into 100ml 33.3% mango juice without pulp | • Adding 0.7g thickener into 100ml 33.3% mango juice without pulp | • Adding 0.3g thickener into 100ml 33.3% mango juice without pulp | • Using 100ml 33.3% mango juice without pulp |
| | Orange juice | • Adding 3.0g thickener into 100ml 100% orange juice without pulp | • Adding 1.4g thickener into 100ml 100% orange juice without pulp | • Adding 0.7g thickener into 100ml 100% orange juice without pulp | • Adding 0.4g thickener into 100ml 100% orange juice without pulp | • Using 100ml 100% orange juice without pulp |
| | Papaya juice | • Blending the papaya with water in 1:2 ratio and filtering away the residue to obtain 50% papaya juice | | | | |
| | | • Adding 2.8g thickener into 100ml 50% papaya juice | • Adding 1.4g thickener into 100ml 50% papaya juice | • Adding 0.7g thickener into 100ml 50% papaya juice | • Adding 0.4g thickener into 100ml 50% papaya juice | • Using 100ml 50% papaya juice |
| | Pineapple juice | • Adding 4.2g thickener into 100ml 100% pineapple juice without pulp | • Adding 1.4g thickener into 100ml 100% pineapple juice without pulp | • Adding 0.7g thickener into 120ml 100% pineapple juice without pulp | • Adding 0.4g thickener into 100ml 100% pineapple juice without pulp | • Using 100ml 100% pineapple juice without pulp |
| | Watermelon juice | • Adding 4.2g thickener into 100ml 100% watermelon juice without pulp | • Adding 2.1g thickener into 100ml 100% apple juice without pulp | • Adding 1.4g thickener into 120ml 100% watermelon juice without pulp | • Adding 0.7g thickener into 100ml 100% watermelon juice without pulp | • Using 100ml 100% watermelon juice without pulp |

<table>
<tr><td rowspan="3"></td><td>Soybean milk</td><td>• Adding 2.1g thickener into 100ml prepacked no added sugar soybean milk</td><td>• Adding 1.4g thickener into100ml prepacked no added sugar soybean milk</td><td>• Adding 0.7g thickener into100ml prepacked no added sugar soybean milk</td><td>• Adding 0.35g thickener into 100ml prepacked no added sugar soybean milk</td><td>• Using 100ml prepacked no added sugar soybean milk</td></tr>
<tr><td rowspan="2">Coconut milk</td><td colspan="5">• Boiling 400ml coconut milk, 1.4L water and 15g sugar with medium heat for 5 mins</td></tr>
<tr><td>• Adding 4.2g thickener into 100ml coconut milk</td><td>• Adding 1.4g thickener into 100ml coconut milk</td><td>• Adding 0.7g thickener into 100ml coconut milk</td><td>• Adding 0.3g thickener into 100ml coconut milk</td><td>• Using 100ml coconut milk</td></tr>
<tr><td rowspan="6">Thickened by corn starch</td><td rowspan="2">Black bean sauce</td><td colspan="5">• Adding 10g salted black beans, 10ml soy sauce and 10ml oil into 100 ml water<br>• Boiling for 1 min with medium heat</td></tr>
<tr><td>• Dissolving 10g corn starch into 100 ml water<br>• Adding corn starch solution slowly and boiling with medium heat for 4 mins<br>• Filtering away the preserved beans and excess lump</td><td>• Dissolving 5g corn starch into 100 ml water<br>• Adding corn starch solution slowly and boiling with medium heat for 4 mins<br>• Filtering away the preserved beans and excess lump</td><td>• Dissolving 3.5g corn starch into 100 ml water<br>• Adding corn starch solution slowly and boiling with medium heat for 4 mins<br>• Filtering away the preserved beans and excess lump</td><td>• Dissolving 2.5g corn starch into 100 ml water<br>• Adding corn starch solution slowly and boiling with medium heat for 4 mins<br>• Filtering away the preserved beans and excess lump</td><td>• Filter away the preserved beans and excess lump</td></tr>
<tr><td rowspan="2">Chu hou paste<br>*Level 4 is too sticky so it is not recommended to be eaten individually</td><td colspan="5">• Adding 5 g chu hou paste into 100 ml water and stirring to make it dissolve completely<br>• Boiling for 1.25 mins with medium heat</td></tr>
<tr><td>• Dissolving 12g corn starch into 100 ml water<br>• Adding corn starch solution slowly and boiling with medium heat for 1.25 mins</td><td>• Dissolving 10g corn starch into 100 ml water<br>• Adding 80ml corn starch solution slowly and boiling with medium heat for 1.25 mins</td><td>• Dissolving 10g corn starch into 100 ml water<br>• Adding 50ml corn starch solution slowly and boiling with medium heat for 1.25 mins</td><td>• Dissolving 5g corn starch into 100 ml water<br>• Adding corn starch solution slowly and boiling with medium heat for 1.25 mins</td><td></td></tr>
<tr><td rowspan="2">Coconut curry sauce</td><td colspan="5">• Adding 5g coconut curry sauce into 100ml water and stirring to make it dissolve completely<br>• Boiling for 1.25 mins with medium heat</td></tr>
<tr><td>• Dissolving 12.5g corn starch into 100 ml water</td><td>• Dissolving 7.5g corn starch into 100 ml water</td><td>• Dissolving 5g corn starch into 100 ml water</td><td>• Dissolving 3.5g corn starch into 100 ml water</td><td>• Dissolving 2g corn starch into 100 ml water</td></tr>
</table>

| | | | | | | |
|---|---|---|---|---|---|---|
| | | • Adding corn starch solution slowly and boiling with medium heat for 1.25 mins | • Adding corn starch solution slowly and boiling with medium heat for 1.25 mins | • Adding corn starch solution slowly and boiling with medium heat for 1.25 mins | • Adding corn starch solution slowly and boiling with medium heat for 1.25 mins | • Adding corn starch solution slowly and boil with medium heat for 1.25 mins |
| | Red fermented bean curd sauce | • Adding 5 g red fermented bean curd into 100 ml water and stirring to make it dissolve completely<br>• Boiling for 1.25 mins with medium heat | | | | |
| | | • Dissolving 10g corn starch into 100 ml water<br>• Adding corn starch solution slowly and boiling with medium heat for 1.25 mins | • Dissolving 7.5g corn starch into 100 ml water<br>• Adding corn starch solution slowly and boiling with medium heat for 1.25 mins | • Dissolving 5g corn starch into 100 ml water<br>• Adding corn starch solution slowly and boiling with medium heat for 1.25 mins | | |
| No thickener added | Rice<br>**Level 4 rice/congee is too sticky so it is not recommended to be eaten individually* | • Soaking rice with water in 1:2 ratio for 1 hr<br>• Cooking the rice<br>• Blending 200g rice with 75g water | • Soaking rice with water in 1:13.3 ratio for 1 hr<br>• Steaming the rice with water in cooker for 30 mins and blending the rice | • Soaking rice with water in 1:20 ratio for 1 hr<br>• Steaming the rice with water in cooker for 30 mins and blending the rice | • Soaking rice with water in 1:25 ratio for 1 hr<br>• Steaming the rice with water in cooker for 30 mins and blending the rice | |
| | Peanut sauce<br>**Level 3 is too sticky so it is not recommended to be eaten individually* | | • Using the commercial peanut sauce | | | |
| | Scallion oil | | | • Using commercial oil<br>• Heating with medium heat for 1.5 mins | | |

**Table S6.** Hardness, adhesiveness and cohesiveness of different food ingredients in different IDDSI level

| | IDDSI Level | | 7 | 6 | 5 | 4 (solid) |
|---|---|---|---|---|---|---|
| Beef | Hardness ($10^3 N/m^2$) | Mean | 78.552 | 17.651 | 10.645 | 3.523 |
| | | SD | 15.928 | 7.003 | 2.022 | 0.621 |
| | Adhesiveness(g·sec) | Mean | 0.000 | 0.000 | 0.000 | -22.635 |
| | | SD | 0.000 | 0.000 | 0.000 | 2.161 |
| | Cohesiveness | Mean | 0.647 | 0.618 | 0.696 | 0.593 |
| | | SD | 0.009 | 0.018 | 0.119 | 0.042 |
| Chicken | Hardness ($10^3 N/m^2$) | Mean | 99.868 | 26.925 | 12.149 | 1.672 |
| | | SD | 42.324 | 7.803 | 2.296 | 0.099 |
| | Adhesiveness(g·sec) | Mean | 0.000 | 0.000 | -1.656 | -54.476 |
| | | SD | 0.000 | 0.000 | 1.871 | 7.506 |
| | Cohesiveness | Mean | 0.509 | 0.609 | 0.511 | 0.534 |
| | | SD | 0.054 | 0.057 | 0.021 | 0.028 |
| Dace paste | Hardness ($10^3 N/m^2$) | Mean | 131.756 | 48.525 | 8.223 | 0.953 |
| | | SD | 13.763 | 6.110 | 0.650 | 0.400 |
| | Adhesiveness(g·sec) | Mean | -3.617 | -8.027 | -4.609 | -23.732 |
| | | SD | 4.343 | 3.415 | 3.329 | 2.493 |
| | Cohesiveness | Mean | 0.733 | 0.758 | 0.514 | 0.743 |
| | | SD | 0.018 | 0.009 | 0.023 | 0.055 |
| Fish fillets | Hardness ($10^3 N/m^2$) | Mean | 53.970 | 14.489 | 12.528 | 0.849 |
| | | SD | 9.938 | 2.702 | 2.447 | 0.111 |
| | Adhesiveness(g·sec) | Mean | -25.063 | -5.923 | -2.899 | -26.041 |
| | | SD | 42.382 | 6.440 | 1.093 | 3.384 |
| | Cohesiveness | Mean | 0.361 | 0.554 | 0.603 | 0.693 |
| | | SD | 0.048 | 0.047 | 0.023 | 0.023 |
| Pork | Hardness ($10^3 N/m^2$) | Mean | 90.839 | 22.580 | 10.291 | 1.961 |
| | | SD | 20.079 | 6.093 | 2.025 | 0.186 |
| | Adhesiveness(g·sec) | Mean | -6.372 | 0.000 | -2.864 | -58.188 |
| | | SD | 8.991 | 0.000 | 1.433 | 19.393 |
| | Cohesiveness | Mean | 0.652 | 0.669 | 0.530 | 0.606 |
| | | SD | 0.033 | 0.070 | 0.028 | 0.048 |
| Black fungus | Hardness ($10^3 N/m^2$) | Mean | 69.566 | 8.792 | 5.753 | 0.790 |
| | | SD | 16.699 | 5.002 | 1.845 | 0.047 |
| | Adhesiveness(g·sec) | Mean | -5.640 | -18.926 | -6.788 | -25.810 |
| | | SD | 5.160 | 10.870 | 7.597 | 0.415 |
| | Cohesiveness | Mean | 0.830 | 0.724 | 0.458 | 0.892 |
| | | SD | 0.051 | 0.089 | 0.031 | 0.019 |
| Broccoli | Hardness ($10^3 N/m^2$) | Mean | 112.122 | 20.482 | 12.173 | 1.160 |
| | | SD | 28.455 | 5.909 | 0.609 | 0.079 |
| | Adhesiveness(g·sec) | Mean | -15.340 | -3.947 | -5.020 | -48.633 |
| | | SD | 12.793 | 3.636 | 0.784 | 8.398 |
| | Cohesiveness | Mean | 0.091 | 0.214 | 0.199 | 0.752 |
| | | SD | 0.019 | 0.081 | 0.010 | 0.020 |
| Carrot | Hardness ($10^3 N/m^2$) | Mean | 328.473 | 49.487 | 10.267 | 2.194 |
| | | SD | 65.440 | 0.293 | 2.512 | 0.104 |
| | Adhesiveness(g·sec) | Mean | -120.475 | -12.130 | -1.160 | -21.526 |
| | | SD | 66.989 | 9.170 | 0.577 | 3.579 |
| | Cohesiveness | Mean | 0.113 | 0.140 | 0.212 | 0.638 |
| | | SD | 0.009 | 0.008 | 0.015 | 0.025 |
| | Hardness ($10^3 N/m^2$) | Mean | 24.396 | 15.956 | 7.587 | 1.039 |

| | | | | | | |
|---|---|---|---|---|---|---|
| Chinese amaranth | | SD | 8.679 | 8.652 | 1.916 | 0.098 |
| | Adhesiveness(g·sec) | Mean | -28.943 | -2.732 | -10.197 | -40.059 |
| | | SD | 47.158 | 1.814 | 4.683 | 10.482 |
| | Cohesiveness | Mean | 0.261 | 0.282 | 0.326 | 0.735 |
| | | SD | 0.112 | 0.056 | 0.058 | 0.028 |
| Chinese white cabbage | Hardness ($10^3 N/m^2$) | Mean | 53.750 | 20.218 | 12.438 | 0.769 |
| | | SD | 12.412 | 5.645 | 1.362 | 0.073 |
| | Adhesiveness(g·sec) | Mean | -4.851 | -1.216 | -22.660 | -36.051 |
| | | SD | 1.615 | 1.296 | 5.963 | 3.356 |
| | Cohesiveness | Mean | 0.256 | 0.322 | 0.434 | 0.749 |
| | | SD | 0.045 | 0.082 | 0.033 | 0.005 |
| Choy sum | Hardness ($10^3 N/m^2$) | Mean | 346.530 | 38.179 | 17.082 | 0.523 |
| | | SD | 80.989 | 13.928 | 4.704 | 0.043 |
| | Adhesiveness(g·sec) | Mean | -5.629 | -4.777 | -3.694 | -17.096 |
| | | SD | 3.596 | 5.334 | 0.341 | 2.695 |
| | Cohesiveness | Mean | 0.156 | 0.230 | 0.508 | 0.688 |
| | | SD | 0.018 | 0.130 | 0.074 | 0.027 |
| Eggplant | Hardness ($10^3 N/m^2$) | Mean | 55.369 | 28.533 | 11.837 | 0.865 |
| | | SD | 20.703 | 8.020 | 1.618 | 0.014 |
| | Adhesiveness(g·sec) | Mean | -70.762 | -13.477 | -14.580 | -48.714 |
| | | SD | 54.458 | 4.458 | 17.278 | 8.026 |
| | Cohesiveness | Mean | 0.166 | 0.171 | 0.378 | 0.771 |
| | | SD | 0.036 | 0.013 | 0.031 | 0.010 |
| Hairy gourd | Hardness ($10^3 N/m^2$) | Mean | 164.514 | 45.008 | 8.881 | 0.672 |
| | | SD | 28.208 | 2.610 | 0.988 | 0.056 |
| | Adhesiveness(g·sec) | Mean | -40.535 | -4.395 | -5.848 | -29.658 |
| | | SD | 43.299 | 1.068 | 1.676 | 7.569 |
| | Cohesiveness | Mean | 0.109 | 0.085 | 0.181 | 0.774 |
| | | SD | 0.011 | 0.016 | 0.015 | 0.055 |
| Iceberg lettuce | Hardness ($10^3 N/m^2$) | Mean | 78.350 | 36.350 | 9.153 | 1.476 |
| | | SD | 19.540 | 6.523 | 0.855 | 0.214 |
| | Adhesiveness(g·sec) | Mean | -5.502 | -2.559 | -16.699 | -16.128 |
| | | SD | 6.126 | 2.416 | 5.972 | 3.335 |
| | Cohesiveness | Mean | 0.368 | 0.356 | 0.349 | 0.698 |
| | | SD | 0.019 | 0.025 | 0.034 | 0.054 |
| Indian lettuce | Hardness ($10^3 N/m^2$) | Mean | 76.838 | 32.388 | 15.432 | 0.513 |
| | | SD | 30.740 | 10.448 | 5.173 | 0.044 |
| | Adhesiveness(g·sec) | Mean | -0.943 | -1.135 | -6.924 | -20.099 |
| | | SD | 0.251 | 0.842 | 8.160 | 2.711 |
| | Cohesiveness | Mean | 0.326 | 0.403 | 0.448 | 0.750 |
| | | SD | 0.041 | 0.035 | 0.052 | 0.041 |
| Mini Tientsin cabbage | Hardness ($10^3 N/m^2$) | Mean | 111.814 | 25.254 | 16.660 | 1.003 |
| | | SD | 13.419 | 8.584 | 2.119 | 0.041 |
| | Adhesiveness(g·sec) | Mean | -13.969 | -2.053 | -4.703 | -49.820 |
| | | SD | 8.539 | 0.885 | 1.663 | 3.703 |
| | Cohesiveness | Mean | 0.129 | 0.360 | 0.366 | 0.793 |
| | | SD | 0.041 | 0.080 | 0.020 | 0.011 |
| Shiitake mushroom | Hardness ($10^3 N/m^2$) | Mean | 135.528 | 34.425 | 5.030 | 0.704 |
| | | SD | 26.478 | 11.244 | 0.088 | 0.034 |
| | Adhesiveness(g·sec) | Mean | 0.000 | -3.454 | -10.142 | -32.921 |
| | | SD | 0.000 | 3.671 | 2.559 | 0.797 |
| | Cohesiveness | Mean | 0.639 | 0.638 | 0.370 | 0.643 |
| | | SD | 0.017 | 0.055 | 0.008 | 0.022 |

| | | | | | | |
|---|---|---|---|---|---|---|
| Snow Fungus | Hardness ($10^3 N/m^2$) | Mean | 10.995 | 1.971 | 1.656 | 0.894 |
| | | SD | 6.118 | 0.165 | 0.131 | 0.024 |
| | Adhesiveness(g·sec) | Mean | -46.031 | -26.181 | -12.615 | -35.083 |
| | | SD | 23.895 | 18.529 | 2.816 | 1.682 |
| | Cohesiveness | Mean | 0.741 | 0.391 | 0.550 | 0.851 |
| | | SD | 0.103 | 0.170 | 0.022 | 0.016 |
| Tomato | Hardness ($10^3 N/m^2$) | Mean | 64.593 | 28.437 | 7.292 | 0.817 |
| | | SD | 8.844 | 7.939 | 1.492 | 0.066 |
| | Adhesiveness(g·sec) | Mean | -24.316 | -8.073 | -4.447 | -24.094 |
| | | SD | 6.575 | 2.954 | 1.430 | 10.602 |
| | Cohesiveness | Mean | 0.257 | 0.260 | 0.349 | 0.732 |
| | | SD | 0.068 | 0.031 | 0.029 | 0.021 |
| Wax gourd | Hardness ($10^3 N/m^2$) | Mean | 12.900 | 9.859 | 7.759 | 0.548 |
| | | SD | 0.945 | 0.705 | 1.765 | 0.016 |
| | Adhesiveness(g·sec) | Mean | -17.309 | -6.696 | -10.295 | -22.272 |
| | | SD | 2.271 | 1.849 | 7.759 | 1.664 |
| | Cohesiveness | Mean | 0.161 | 0.195 | 0.186 | 0.677 |
| | | SD | 0.018 | 0.029 | 0.029 | 0.025 |
| White radish | Hardness ($10^3 N/m^2$) | Mean | 392.504 | 37.061 | 12.306 | 0.859 |
| | | SD | 22.507 | 5.951 | 1.410 | 0.055 |
| | Adhesiveness(g·sec) | Mean | -6.941 | -2.452 | -1.740 | -50.691 |
| | | SD | 1.585 | 3.482 | 0.749 | 3.193 |
| | Cohesiveness | Mean | 0.078 | 0.140 | 0.198 | 0.774 |
| | | SD | 0.006 | 0.028 | 0.006 | 0.035 |

| | | IDDSI Level | 7 | | 6 | 5 | 4(solid) |
|---|---|---|---|---|---|---|---|
| | | | Raw | Cooked | | | |
| Apple | Hardness ($10^3 N/m^2$) | Mean | 347.147 | 272.910 | 47.120 | 9.523 | 4.841 |
| | | SD | 88.209 | 104.096 | 6.572 | 1.812 | 0.541 |
| | Adhesiveness(g·sec) | Mean | -9.461 | -84.404 | -16.373 | -17.242 | -18.520 |
| | | SD | 10.088 | 34.152 | 8.846 | 17.433 | 4.787 |
| | Cohesiveness | Mean | 0.164 | 0.080 | 0.152 | 0.285 | 0.587 |
| | | SD | 0.04201 | 0.029 | 0.022 | 0.038 | 0.065 |
| Kiwi | Hardness ($10^3 N/m^2$) | Mean | | 69.603 | 37.143 | 1.565 | 0.451 |
| | | SD | | 12.395 | 5.368 | 0.265 | 0.018 |
| | Adhesiveness(g·sec) | Mean | | -50.563 | -20.655 | -11.108 | -19.499 |
| | | SD | | 18.086 | 7.174 | 2.373 | 1.108 |
| | Cohesiveness | Mean | | 0.111 | 0.116 | 0.477 | 0.715 |
| | | SD | | 0.019 | 0.023 | 0.036 | 0.026 |
| Mango | Hardness ($10^3 N/m^2$) | Mean | | 40.275 | 17.839 | 2.250 | 0.817 |
| | | SD | | 3.009 | 6.057 | 0.355 | 0.066 |
| | Adhesiveness(g·sec) | Mean | | -20.052 | -5.817 | -19.580 | -35.838 |
| | | SD | | 9.498 | 5.774 | 5.397 | 2.872 |
| | Cohesiveness | Mean | | 0.110 | 0.124 | 0.486 | 0.760 |
| | | SD | | 0.011 | 0.043 | 0.044 | 0.023 |
| Orange | Hardness ($10^3 N/m^2$) | Mean | | 43.309 | 34.564 | 5.174 | 1.388 |
| | | SD | | 4.595 | 1.607 | 1.013 | 0.104 |
| | Adhesiveness(g·sec) | Mean | | -7.891 | -7.781 | -2.930 | -17.698 |
| | | SD | | 4.007 | 4.242 | 1.828 | 8.082 |
| | Cohesiveness | Mean | | 0.263 | 0.303 | 0.400 | 0.613 |
| | | SD | | 0.059 | 0.078 | 0.013 | 0.093 |
| Papaya | Hardness ($10^3 N/m^2$) | Mean | | 102.333 | 25.186 | 4.473 | 0.965 |
| | | SD | | 5.513 | 3.613 | 0.253 | 0.023 |
| | Adhesiveness(g·sec) | Mean | | -11.325 | -6.336 | -4.857 | -42.201 |
| | | SD | | 2.592 | 5.177 | 1.481 | 7.377 |
| | Cohesiveness | Mean | | 0.177 | 0.174 | 0.238 | 0.764 |
| | | SD | | 0.026 | 0.016 | 0.023 | 0.026 |
| Pineapple | Hardness ($10^3 N/m^2$) | Mean | 128.500 | 92.584 | 28.961 | 13.922 | 1.101 |
| | | SD | 20.210 | 24.380 | 7.504 | 2.149 | 0.036 |
| | Adhesiveness(g·sec) | Mean | -7.310 | -12.709 | -4.285 | -13.299 | -39.812 |
| | | SD | 4.76022 | 4.560 | 4.345 | 12.584 | 8.219 |
| | Cohesiveness | Mean | 0.160 | 0.129 | 0.209 | 0.229 | 0.776 |
| | | SD | 0.04052 | 0.025 | 0.087 | 0.029 | 0.064 |
| Watermelon | Hardness ($10^3 N/m^2$) | Mean | | 138.908 | 24.957 | 6.925 | 0.634 |
| | | SD | | 36.304 | 10.148 | 1.184 | 0.052 |
| | Adhesiveness(g·sec) | Mean | | -7.941 | -2.351 | -1.607 | -19.412 |
| | | SD | | 6.291 | 1.232 | 1.249 | 2.056 |
| | Cohesiveness | Mean | | 0.140 | 0.241 | 0.229 | 0.748 |
| | | SD | | 0.015 | 0.027 | 0.031 | 0.033 |

| | | IDDSI Level | 7 | 6 | 5 | 4(solid) | |
|---|---|---|---|---|---|---|---|
| Century egg | Hardness ($10^3N/m^2$) | Mean | 26.892 | 6.810 | 2.978 | 2.055 | |
| | | SD | 5.989 | 1.155 | 0.175 | 0.438 | |
| | Adhesiveness(g·sec) | Mean | -1.265 | -14.387 | -24.422 | -53.032 | |
| | | SD | 0.704 | 18.917 | 2.605 | 11.414 | |
| | Cohesiveness | Mean | 0.792 | 0.695 | 0.571 | 0.710 | |
| | | SD | 0.062 | 0.054 | 0.031 | 0.074 | |
| Dried bean curd | Hardness ($10^3N/m^2$) | Mean | 5.176 | 3.700 | 3.068 | 0.672 | |
| | | SD | 3.766 | 0.273 | 0.111 | 0.024 | |
| | Adhesiveness(g·sec) | Mean | -7.025 | -3.740 | -6.098 | -34.446 | |
| | | SD | 12.482 | 1.047 | 3.329 | 1.576 | |
| | Cohesiveness | Mean | 0.550 | 0.585 | 0.404 | 0.743 | |
| | | SD | 0.065 | 0.030 | 0.022 | 0.024 | |
| Dried octopus | Hardness ($10^3N/m^2$) | Mean | 20.462 | 15.275 | 0.886 | NA | |
| | | SD | 3.948 | 3.871 | 0.118 | NA | |
| | Adhesiveness(g·sec) | Mean | -32.751 | -23.155 | -16.773 | NA | |
| | | SD | 5.702 | 18.662 | 4.890 | NA | |
| | Cohesiveness | Mean | 0.610 | 0.604 | 0.625 | NA | |
| | | SD | 0.051 | 0.041 | 0.081 | NA | |
| Dried shrimp | Hardness ($10^3N/m^2$) | Mean | 126.230 | 46.983 | 9.050 | 3.936 | |
| | | SD | 33.994 | 5.293 | 4.031 | 0.759 | |
| | Adhesiveness(g·sec) | Mean | -17.610 | -8.703 | -18.810 | -15.718 | |
| | | SD | 11.819 | 10.181 | 12.310 | 3.486 | |
| | Cohesiveness | Mean | 0.594 | 0.538 | 0.382 | 0.533 | |
| | | SD | 0.033 | 0.090 | 0.040 | 0.016 | |
| Egg | Hardness ($10^3N/m^2$) | Mean | 23.380 | 8.796 | 7.128 | 0.737 | |
| | | SD | 2.429 | 1.074 | 0.511 | 0.184 | |
| | Adhesiveness(g·sec) | Mean | -1.327 | 0.000 | -3.062 | -5.506 | |
| | | SD | 1.225 | 0.000 | 2.443 | 2.159 | |
| | Cohesiveness | Mean | 0.575 | 0.736 | 0.548 | 0.637 | |
| | | SD | 0.055 | 0.092 | 0.054 | 0.044 | |
| Tofu | Hardness ($10^3N/m^2$) | Mean | 370.102 | 20.380 | 9.007 | 1.008 | 1.031* |
| | | SD | 32.947 | 5.406 | 1.409 | 0.139 | 0.074* |
| | Adhesiveness(g·sec) | Mean | 0.000 | -2.772 | -4.347 | -42.301 | -11.815* |
| | | SD | 0.000 | 1.591 | 2.554 | 7.003 | 2.496* |
| | Cohesiveness | Mean | 0.619 | 0.541 | 0.620 | 0.738 | 0.517* |
| | | SD | 0.032 | 0.027 | 0.074 | 0.043 | 0.034* |

*Data from the pre-packaged tofu fa

| | IDDSI Level | | 7 | 6 | 5 | 4 (solid) |
|---|---|---|---|---|---|---|
| Chestnut | Hardness ($10^3 N/m^2$) | Mean | 158.979 | 43.334 | 18.850 | 3.027 |
| | | SD | 60.241 | 18.025 | 2.304 | 0.499 |
| | Adhesiveness(g·sec) | Mean | -6.048 | -15.701 | -28.977 | -89.159 |
| | | SD | 7.884 | 17.258 | 24.224 | 11.644 |
| | Cohesiveness | Mean | 0.123 | 0.195 | 0.149 | 0.681 |
| | | SD | 0.084 | 0.034 | 0.017 | 0.051 |
| Corn | Hardness ($10^3 N/m^2$) | Mean | 33.802 | | 12.073 | 0.605 |
| | | SD | 10.062 | | 2.191 | 0.026 |
| | Adhesiveness(g·sec) | Mean | -0.939 | | -3.234 | -18.592 |
| | | SD | 0.735 | | 0.686 | 2.181 |
| | Cohesiveness | Mean | 0.323 | | 0.301 | 0.750 |
| | | SD | 0.125 | | 0.032 | 0.017 |
| Green bean vermicelli | Hardness ($10^3 N/m^2$) | Mean | 5.092 | 3.145 | 3.749 | 1.114 |
| | | SD | 1.789 | 0.160 | 0.575 | 0.101 |
| | Adhesiveness(g·sec) | Mean | -9.632 | -2.328 | -4.330 | -59.225 |
| | | SD | 15.444 | 0.767 | 2.683 | 5.303 |
| | Cohesiveness | Mean | 0.528 | 0.371 | 0.302 | 0.728 |
| | | SD | 0.100 | 0.040 | 0.063 | 0.010 |
| Potato | Hardness ($10^3 N/m^2$) | Mean | 165.212 | 45.047 | 12.005 | 2.361 |
| | | SD | 44.039 | 14.699 | 2.948 | 0.374 |
| | Adhesiveness(g·sec) | Mean | -66.825 | -24.478 | -8.854 | -73.741 |
| | | SD | 51.058 | 3.613 | 5.954 | 7.647 |
| | Cohesiveness | Mean | 0.088 | 0.114 | 0.187 | 0.840 |
| | | SD | 0.018 | 0.009 | 0.023 | 0.032 |
| Pumpkin | Hardness ($10^3 N/m^2$) | Mean | 287.067 | 36.022 | 9.964 | 1.494 |
| | | SD | 129.145 | 11.928 | 1.242 | 0.067 |
| | Adhesiveness(g·sec) | Mean | -99.244 | -39.719 | -21.735 | -59.596 |
| | | SD | 85.871 | 28.689 | 11.040 | 5.538 |
| | Cohesiveness | Mean | 0.286 | 0.245 | 0.267 | 0.752 |
| | | SD | 0.142 | 0.026 | 0.020 | 0.046 |
| Rice | Hardness ($10^3 N/m^2$) | Mean | 49.485 | 37.167 | 16.219 | 1.837 |
| | | SD | 6.279 | 9.125 | 1.012 | 0.176 |
| | Adhesiveness(g·sec) | Mean | -35.531 | -75.952 | -91.718 | -39.924 |
| | | SD | 18.443 | 60.964 | 75.568 | 2.883 |
| | Cohesiveness | Mean | 0.669 | 0.634 | 0.528 | 0.816 |
| | | SD | 0.072 | 0.033 | 0.034 | 0.011 |
| Taro | Hardness ($10^3 N/m^2$) | Mean | 216.213 | 43.512 | 9.038 | 1.575 |
| | | SD | 43.871 | 5.919 | 1.394 | 0.051 |
| | Adhesiveness(g·sec) | Mean | -165.118 | -20.903 | -25.182 | -48.243 |
| | | SD | 63.665 | 11.070 | 9.096 | 3.120 |
| | Cohesiveness | Mean | 0.140 | 0.228 | 0.241 | 0.878 |
| | | SD | 0.039 | 0.021 | 0.012 | 0.038 |

**Table S7.** Hardness, adhesiveness and cohesiveness of other dishes and commercial products in different IDDSI level.

| | IDDSI Level | | 7 | 6 | 5 | 4(soft meal) | 4(puree) |
|---|---|---|---|---|---|---|---|
| Fried dace with salted black beans | Hardness ($10^3N/m^2$) | Mean | 303.801 | 26.309 | 16.005 | 4.915 | 0.719 |
| | | SD | 123.357 | 2.149 | 2.902 | 0.422 | 0.033 |
| | Adhesiveness(g·sec) | Mean | -62.144 | -4.180 | -18.729 | -11.379 | -20.117 |
| | | SD | 68.463 | 0.772 | 8.754 | 6.062 | 3.201 |
| | Cohesiveness | Mean | 0.607 | 0.468 | 0.304 | 0.571 | 0.616 |
| | | SD | 0.099 | 0.030 | 0.032 | 0.014 | 0.033 |
| Fried grouper | Hardness ($10^3N/m^2$) | Mean | 122.160 | 47.756 | 9.697 | 4.058 | 1.209 |
| | | SD | 52.538 | 18.292 | 1.245 | 0.257 | 0.042 |
| | Adhesiveness(g.sec) | Mean | 0.000 | -24.912 | -33.290 | -11.697 | -30.718 |
| | | SD | 0.000 | 28.067 | 11.369 | 5.316 | 3.883 |
| | Cohesiveness | Mean | 0.746 | 0.663 | 0.463 | 0.434 | 0.647 |
| | | SD | 0.056 | 0.178 | 0.032 | 0.019 | 0.046 |
| Steamed snubnose pompano | Hardness ($10^3N/m^2$) | Mean | 34.837 | 8.657 | 7.305 | 3.050 | 2.442 |
| | | SD | 5.935 | 1.955 | 0.482 | 0.680 | 0.277 |
| | Adhesiveness(g·sec) | Mean | -12.404 | -3.138 | -14.575 | -20.164 | -66.395 |
| | | SD | 7.722 | 0.571 | 4.072 | 13.731 | 9.092 |
| | Cohesiveness | Mean | 0.412 | 0.405 | 0.396 | 0.517 | 0.743 |
| | | SD | 0.045 | 0.029 | 0.047 | 0.073 | 0.015 |
| Barbecue pork | Hardness ($10^3N/m^2$) | Mean | 151.067 | 25.979 | 17.391 | 3.710 | 1.893 |
| | | SD | 42.324 | 9.904 | 3.452 | 1.308 | 0.386 |
| | Adhesiveness(g·sec) | Mean | -5.691 | -5.917 | -12.574 | -9.247 | -39.156 |
| | | SD | 7.147 | 7.266 | 5.045 | 6.478 | 7.744 |
| | Cohesiveness | Mean | 0.451 | 0.481 | 0.364 | 0.407 | 0.490 |
| | | SD | 0.036 | 0.085 | 0.024 | 0.072 | 0.003 |
| Sweet and sour pork | Hardness ($10^3N/m^2$) | Mean | 85.676 | | 18.943 | 2.160 | 0.929 |
| | | SD | 21.873 | | 2.331 | 0.556 | 0.062 |
| | Adhesiveness(g·sec) | Mean | -4.310 | | -13.446 | -9.105 | -24.487 |
| | | SD | 2.708 | | 10.865 | 3.954 | 3.877 |
| | Cohesiveness | Mean | 0.082 | | 0.382 | 0.885 | 0.989 |
| | | SD | 0.040 | | 0.041 | 0.037 | 0.006 |
| Trotter | Hardness ($10^3N/m^2$) | Mean | 91.790 | 9.415 | 1.321 | 3.308 | 4.552 |
| | | SD | 53.815 | 0.956 | 0.162 | 1.116 | 1.964 |
| | Adhesiveness(g·sec) | Mean | -223.645 | -23.603 | -26.005 | -14.600 | -11.144 |
| | | SD | 174.760 | 25.434 | 14.385 | 4.514 | 7.575 |
| | Cohesiveness | Mean | 0.622 | 0.590 | 0.582 | 0.668 | 0.917 |
| | | SD | 0.108 | 0.055 | 0.095 | 0.035 | 0.026 |
| Garlic | Hardness ($10^3N/m^2$) | Mean | 17.117 | | 6.751 | | 3.178 |
| | | SD | 8.794 | | 0.897 | | 0.164 |
| | Adhesiveness(g·sec) | Mean | -29.937 | | -15.256 | | -45.171 |
| | | SD | 10.206 | | 3.042 | | 11.400 |
| | Cohesiveness | Mean | 0.296 | | 0.256 | | 0.734 |
| | | SD | 0.136 | | 0.009 | | 0.119 |
| Peanut | Hardness ($10^3N/m^2$) | Mean | 108.188 | | | | 0.925 |
| | | SD | 9.514 | | | | 0.107 |
| | Adhesiveness(g·sec) | Mean | -3.236 | | | | -49.706 |
| | | SD | 3.111 | | | | 3.170 |
| | Cohesiveness | Mean | 0.276 | | | | 0.697 |
| | | SD | 0.048 | | | | 0.027 |
| Salted black bean | Hardness ($10^3N/m^2$) | Mean | 13.560 | | 9.222 | | 1.571 |

| | | | | | | | |
|---|---|---|---|---|---|---|---|
| | | SD | 4.158 | | 1.775 | | 0.122 |
| | Adhesiveness(g·sec) | Mean | -2.292 | | -17.662 | | -48.843 |
| | | SD | 1.382 | | 14.633 | | 14.733 |
| | Cohesiveness | Mean | 0.486 | | 0.315 | | 0.776 |
| | | SD | 0.068 | | 0.026 | | 0.050 |
| Scallion oil | Hardness ($10^3N/m^2$) | Mean | 26.271 | | 13.842 | | 1.054 |
| | | SD | 10.002 | | 3.101 | | 0.053 |
| | Adhesiveness(g·sec) | Mean | -10.366 | | -17.255 | | -33.972 |
| | | SD | 8.937 | | 10.762 | | 7.013 |
| | Cohesiveness | Mean | 0.347 | | 0.406 | | 0.798 |
| | | SD | 0.048 | | 0.016 | | 0.040 |
| Mooncake | Hardness ($10^3N/m^2$) | Mean | 115.154 | | | 4.608 | |
| | | SD | 35.852 | | | 0.339 | |
| | Adhesiveness(g.sec) | Mean | -15.812 | | | -9.929 | |
| | | SD | 7.985 | | | 6.844 | |
| | Cohesiveness | Mean | 0.136 | | | 0.618 | |
| | | SD | 0.033 | | | 0.047 | |
| Sago | Hardness ($10^3N/m^2$) | Mean | | | 0.888 | | |
| | | SD | | | 0.231 | | |
| | Adhesiveness(g·sec) | Mean | | | -11.680 | | |
| | | SD | | | 7.176 | | |
| | Cohesiveness | Mean | | | 0.646 | | |
| | | SD | | | 0.086 | | |
| Sesame dumpling | Hardness ($10^3N/m^2$) | Mean | 17.012 | | | 1.435 | |
| | | SD | 3.551 | | | 0.153 | |
| | Adhesiveness(g.sec) | Mean | -47.165 | | | -14.619 | |
| | | SD | 7.773 | | | 2.112 | |
| | Cohesiveness | Mean | 0.796 | | | 0.499 | |
| | | SD | 0.012 | | | 0.017 | |
| Dried bonito | Hardness ($10^3N/m^2$) | Mean | 27.224 | 19.302 | 10.752 | | 8.835 |
| | | SD | 9.876 | 2.517 | 0.803 | | 3.670 |
| | Adhesiveness(g·sec) | Mean | -61.954 | -58.111 | -2.232 | | -7.833 |
| | | SD | 82.394 | 27.129 | 1.369 | | 3.831 |
| | Cohesiveness | Mean | 0.572 | 0.596 | 0.617 | | 0.473 |
| | | SD | 0.071 | 0.031 | 0.020 | | 0.007 |
| Preserved Radish | Hardness ($10^3N/m^2$) | Mean | 114.073 | 30.286 | 18.394 | 4.790 | 3.884 |
| | | SD | 42.019 | 10.514 | 3.047 | 0.579 | 0.251 |
| | Adhesiveness(g·sec) | Mean | -20.663 | -2.696 | -9.099 | -12.349 | -31.333 |
| | | SD | 12.198 | 2.283 | 3.826 | 2.451 | 25.948 |
| | Cohesiveness | Mean | 0.631 | 0.521 | 0.516 | 0.357 | 0.500 |
| | | SD | 0.036 | 0.027 | 0.020 | 0.031 | 0.047 |
| Steamed rice roll | Hardness ($10^3N/m^2$) | Mean | 51.670 | 9.049 | | 1.751 | |
| | | SD | 6.488 | 2.270 | | 0.320 | |
| | Adhesiveness(g·sec) | Mean | -28.944 | -3.294 | | -20.339 | |
| | | SD | 40.148 | 2.411 | | 3.702 | |
| | Cohesiveness | Mean | 0.638 | 0.837 | | 0.495 | |
| | | SD | 0.035 | 0.041 | | 0.063 | |

**Table S8.** Result of Flow test and viscosity of different ingredients in different IDDSI levels.

| | IDDSI Level | | 4 (liquid) | 3 | 2 | 1 | 0 |
|---|---|---|---|---|---|---|---|
| Fish soup | Flow test – Remaining volume (ml) | Mean | 10.0 | 8.7 | 4.6 | 2.1 | 0.0 |
| | Viscosity(cP) | Mean | 1101.3 | 275.6 | 95.8 | 47.8 | 1.7 |
| | | SD | 53.5 | 20.0 | 7.3 | 15.7 | 0.0 |
| Pork soup | Flow test – Remaining volume (ml) | Mean | 10.0 | 9.7 | 7.7 | 2.6 | 0.0 |
| | Viscosity(cP) | Mean | 1032.5 | 395.0 | 198.1 | 69.4 | 1.2 |
| | | SD | 41.2 | 47.2 | 10.4 | 7.5 | 0.1 |
| Carrot juice | Flow test – Remaining volume (ml) | Mean | 10.0 | 8.6 | 4.6 | 2.8 | 0.0 |
| | Viscosity (cP) | Mean | 645.5 | 244.0 | 120.2 | 79.9 | 1.4 |
| | | SD | 7.9 | 9.5 | 8.4 | 7.0 | 0.0 |
| Choy sum soup | Flow test – Remaining volume (ml) | Mean | 10.0 | 8.5 | 5.2 | 3.2 | 0.0 |
| | Viscosity (cP) | Mean | 1085.7 | 251.1 | 137.6 | 70.7 | 1.3 |
| | | SD | 45.6 | 25.1 | 8.5 | 12.9 | 0.0 |
| Corn soup | Flow test – Remaining volume (ml) | Mean | 10.0 | 9.3 | 5.7 | 2.9 | 0.0 |
| | Viscosity (cP) | Mean | 813.9 | 327.4 | 132.4 | 66.1 | 6.3 |
| | | SD | 17.1 | 14.4 | 8.3 | 8.4 | 0.2 |
| Pumpkin soup | Flow test – Remaining volume (ml) | Mean | 10.0 | 9.7 | 5.9 | 2.8 | 0.0 |
| | Viscosity (cP) | Mean | 1225.3 | 334.5 | 143.2 | 51.4 | 3.1 |
| | | SD | 67.6 | 19.7 | 2.4 | 8.2 | 0.3 |
| Apple juice | Flow test – Remaining volume (ml) | Mean | 10.0 | 9.6 | 7.6 | 2.2 | 0.0 |
| | Viscosity (cP) | Mean | 1132.0 | 442.1 | 197.8 | 58.8 | 1.5 |
| | | SD | 50.1 | 16.6 | 7.5 | 7.8 | 0.1 |
| Mango juice | Flow test – Remaining volume (ml) | Mean | 10.0 | 9.7 | 7.5 | 3.5 | 0.4 |
| | Viscosity (cP) | Mean | 1271.3 | 349.4 | 164.3 | 72.6 | 21.2 |
| | | SD | 21.2 | 11.5 | 1.9 | 2.9 | 1.0 |
| Orange juice | Flow test – Remaining volume (ml) | Mean | 10.0 | 8.5 | 4.3 | 1.3 | 0.0 |
| | Viscosity (cP) | Mean | 918.4 | 298.8 | 104.3 | 37.3 | 4.4 |
| | | SD | 25.2 | 8.8 | 3.9 | 2.8 | 0.1 |
| Papaya juice | Flow test – Remaining volume (ml) | Mean | 10.0 | 9.4 | 5.5 | 3.6 | 0.0 |
| | Viscosity (cP) | Mean | 945.5 | 446.7 | 169.3 | 70.6 | 10.9 |
| | | SD | 79.3 | 26.8 | 19.9 | 7.5 | 0.9 |
| Pineapple juice | Flow test – Remaining volume (ml) | Mean | 10.0 | 8.7 | 4.6 | 1.5 | 0.0 |
| | Viscosity (cP) | Mean | 1440.0 | 306.6 | 110.3 | 35.4 | 3.8 |
| | | SD | 25.1 | 33.5 | 4.7 | 6.0 | 0.0 |
| Watermelon juice | Flow test – Remaining volume (ml) | Mean | 10.0 | 9.5 | 6.8 | 2.3 | 0.0 |
| | Viscosity (cP) | Mean | 1071.7 | 413.3 | 154.7 | 59.9 | 1.6 |
| | | SD | 79.6 | 11.0 | 10.9 | 5.6 | 0.1 |
| Soybean milk | Flow test – Remaining volume (ml) | Mean | 10.0 | 9.9 | 7.7 | 3.2 | 0.0 |
| | Viscosity (cP) | Mean | 713.1 | 423.2 | 152.5 | 48.2 | 1.9 |
| | | SD | 75.9 | 19.5 | 1.9 | 14.4 | 0.0 |

| | | | | | | | |
|---|---|---|---|---|---|---|---|
| Coconut milk | Flow test – Remaining volume (ml) | Mean | 10.0 | 9.3 | 6.5 | 1.8 | 0.0 |
| | Viscosity (cP) | Mean | 1192.0 | 307.5 | 138.2 | 43.0 | 4.2 |
| | | SD | 8.7 | 19.8 | 7.5 | 8.9 | 0.2 |
| Black bean sauce | Flow test – Remaining volume (ml) | Mean | 10.0 | 9.7 | 4.7 | 2.9 | 0.0 |
| | Viscosity (cP) | Mean | 2625.3 | 377.6 | 91.7 | 41.6 | 13.6 |
| | | SD | 112.3 | 24.7 | 6.1 | 0.7 | 0.5 |
| Chu hou paste | Flow test – Remaining volume (ml) | Mean | 10.0 | 9.5 | 4.9 | 2.1 | |
| | Viscosity (cP) | Mean | 938.9 | 483.3 | 153.8 | 86.9 | |
| | | SD | 29.1 | 28.5 | 6.9 | 7.9 | |
| Coconut curry sauce | Flow test – Remaining volume (ml) | Mean | 10.0 | 9.1 | 6.0 | 1.5 | 0.0 |
| | Viscosity (cP) | Mean | 1810.0 | 274.3 | 133.9 | 57.8 | 14.6 |
| | | SD | 40.5 | 7.9 | 6.1 | 6.6 | 0.3 |
| Red fermented bean curd sauce | Flow test – Remaining volume (ml) | Mean | 10.0 | 8.7 | 4.4 | | |
| | Viscosity (cP) | Mean | 929.6 | 340.4 | 94.2 | | |
| | | SD | 55.1 | 15.9 | 10.4 | | |
| Rice | Flow test – Remaining volume (ml) | Mean | | 8.3 | 4.2 | 2.3 | |
| | Viscosity (cP) | Mean | | 287.7 | 101.0 | 48.2 | |
| | | SD | | 18.1 | 3.7 | 0.3 | |
| Peanut sauce | Flow test – Remaining volume (ml) | Mean | | 9.6 | | | |
| | Viscosity (cP) | Mean | | 882.0 | | | |
| | | SD | | 10.1 | | | |
| Scallion oil | Flow test – Remaining volume (ml) | Mean | | | 4.9 | | |
| | Viscosity (cP) | Mean | | | 57.6 | | |
| | | SD | | | 1.6 | | |
